# Supplementary material for: The role of solar and geomagnetic activity in endothelial activation and inflammation in the NAS cohort
Source: PLoS One. 2022 Jul 26;17(7):e0268700. doi: 10.1371/journal.pone.0268700 (PMC9321765; doi:10.1371/journal.pone.0268700)
Supplement: S1 Table — (DOCX) [file pone.0268700.s001.docx]

Supplementary Information 1

**Table S1.** Most significant moving average window for air pollutants and log β, 2000-2017

| **Outcome** | **Air Pollutant** | **Moving Average**  **Window** | **Slope** | **p-value** |
| --- | --- | --- | --- | --- |
|  | PM2.5 | 28 | 0.012 | 0.00 |
| **sICAM-1** | BC | 28 | 0.138 | 0.00 |
|  | PN | 22 | 0.000 | 0.00 |
|  | Log(β) | 28 | 0.206 | 0.00 |
|  | PM2.5 | 28 | 0.011 | 0.00 |
| **sVCAM-1** | BC | 2 | 0.036 | >0.05 |
|  | PN | 28 | 0.000 | 0.00 |
|  | Log(β) | 28 | 0.279 | 0.00 |
|  | PM2.5 | 5 | 0.004 | >0.05 |
| **CRP** | BC | 3 | 0.096 | >0.05 |
|  | PN | 2 | 0.000 | >0.05 |
|  | Log(β) | 3 | 0.113 | >0.05 |
|  | PM2.5 | 0 | -0.001 | >0.05 |
| **Fibrinogen** | BC | 7 | 0.017 | >0.05 |
|  | PN | 1 | 0.000 | >0.05 |
|  | Log(β) | 0 | -0.026 | >0.05 |
