# Supplementary material for: The role of solar and geomagnetic activity in endothelial activation and inflammation in the NAS cohort
Source: PLoS One. 2022 Jul 26;17(7):e0268700. doi: 10.1371/journal.pone.0268700 (PMC9321765; doi:10.1371/journal.pone.0268700)
Supplement: S2 Table — (DOCX) [file pone.0268700.s002.docx]

Supplementary Information 2

**Table S2.** Distribution of solar, particulate air pollution, and β activity variables, measured between 2000 and 2017

|  | **Mean ± SD** | **Min** | **Max** | **IQR** |
| --- | --- | --- | --- | --- |
| **IMF (nT)** | 5.9±2.9 | 1.8 | 29.2 | 2.8 |
| **Kp Index*** | 18.9±12.1 | 0.0 | 80.9 | 15.9 |
| **Sunspot Number** | 81.5±71.3 | 0.0 | 351.5 | 107.5 |
| **PM_2.5_** **(**$\boldsymbol{\mu}$**g/m^3^)** | 9.4±6.4 | -0.4 | 58.4 | 6.7 |
| **Black Carbon (**$\boldsymbol{\mu}$**g/m^3^)** | 0.74±0.40 | 0.13 | 2.47 | 0.52 |
| **Particle Number (#/cm^3^)** | 20,963±11,852 | 3,447 | 92,400 | 15,798 |
| **Log Particle β Activity****  * Variables are dimensionless; **Bq/m^3^ | -5.0 ±0.3 | -6.2 | -4.1 | 0.44 |
