# Supplementary material for: The role of solar and geomagnetic activity in endothelial activation and inflammation in the NAS cohort
Source: PLoS One. 2022 Jul 26;17(7):e0268700. doi: 10.1371/journal.pone.0268700 (PMC9321765; doi:10.1371/journal.pone.0268700)
Supplement: S3 Table — (DOCX) [file pone.0268700.s003.docx]

Supplementary Information 3

**Table S3.** Percent change (estimate*IQR*100) of sICAM-1 associated per IQR increase (95% CI) of exposure variable

| **Exposure** | **Moving Average** | **Unadjusted** | **PM_2.5_** | **Black Carbon** | **Particle Number** | **Log β Activity** |
| --- | --- | --- | --- | --- | --- | --- |
| **Sunspots(#)** | 0 | 0.45(0.27,0.64) | 0.42(0.23,0.61) | 0.38(0.18,0.57) | 0.27(0.05,0.50) | 0.37(0.18,0.55) |
|  | 1 | 0.47(0.28,0.65) | 0.43(0.24,0.62) | 0.39(0.20,0.59) | 0.29(0.07,0.52) | 0.38(0.19,0.56) |
|  | 7 | 0.52(0.33,0.71) | 0.49(0.29,0.68) | 0.44(0.24,0.64) | 0.34(0.11,0.58) | 0.43(0.23,0.62) |
|  | 14 | 0.55(0.35,0.75) | 0.51(0.31,0.71) | 0.46(0.25,0.67) | 0.36(0.11,0.61) | 0.45(0.25,0.65) |
|  | 21 | 0.57(0.36,0.77) | 0.52(0.31,0.73) | 0.47(0.26,0.69) | 0.37(0.11,0.63) | 0.45(0.24,0.66) |
|  | 28 | 0.57(0.36,0.78) | 0.51(0.30,0.72) | 0.47(0.25,0.69) | 0.37(0.10,0.64) | 0.44(0.23,0.66) |
| **IMF(nT)** | 0 | 2.20(1.43,2.98) | 2.12(1.34,2.90) | 2.22(1.43,3.01) | 2.13(1.34,2.92) | 1.83(1.04,2.61) |
|  | 1 | 3.12(2.20,4.04) | 3.05(2.13,3.97) | 3.07(2.14,4.01) | 2.89(1.94,3.84) | 2.69(1.77,3.61) |
|  | 7 | 6.85(5.42,8.28) | 6.65(5.21,8.08) | 6.72(5.26,8.18) | 6.47(4.93,8.02) | 6.12(4.66,7.58) |
|  | 14 | 8.44(6.80,10.08) | 8.20(6.56,9.84) | 8.22(6.55,9.89) | 8.04(6.21,9.88) | 7.40(5.71,9.09) |
|  | 21 | 9.49(7.74,11.24) | 9.23(7.47,10.98) | 9.24(7.45,11.02) | 9.21(7.23,11.19) | 8.39(6.59,10.18) |
|  | 28 | 10.13(8.34,11.91) | 9.78(7.99,11.56) | 9.89(8.07,11.71) | 10.04(8.00,12.08) | 8.91(7.06,10.75) |
| **Kp Index *** | 0 | 0.12(0.09,0.15) | 0.12(0.08,0.15) | 0.12(0.09,0.15) | 0.12(0.09,0.16) | 0.10(0.07,0.14) |
|  | 1 | 0.14(0.10,0.18) | 0.14(0.10,0.18) | 0.14(0.10,0.18) | 0.14(0.10,0.18) | 0.12(0.09,0.16) |
|  | 7 | 0.26(0.21,0.32) | 0.26(0.20,0.32) | 0.26(0.20,0.32) | 0.25(0.19,0.30) | 0.24(0.18,0.29) |
|  | 14 | 0.34(0.27,0.40) | 0.33(0.27,0.40) | 0.34(0.27,0.40) | 0.31(0.25,0.38) | 0.30(0.24,0.37) |
|  | 21 | 0.38(0.31,0.45) | 0.38(0.31,0.45) | 0.38(0.31,0.45) | 0.36(0.29,0.43) | 0.35(0.28,0.42) |
|  | 28 | 0.41(0.34,0.49) | 0.41(0.34,0.48) | 0.41(0.34,0.48) | 0.40(0.32,0.47) | 0.37(0.30,0.45) |
