# Supplementary material for: The role of solar and geomagnetic activity in endothelial activation and inflammation in the NAS cohort
Source: PLoS One. 2022 Jul 26;17(7):e0268700. doi: 10.1371/journal.pone.0268700 (PMC9321765; doi:10.1371/journal.pone.0268700)
Supplement: S4 Table — (DOCX) [file pone.0268700.s004.docx]

Supplementary Information 4

**Table S4.** Percent change (estimate*IQR*100) of sVCAM-1 associated per IQR increase (95% CI) of exposure variable

| **Exposure** | **Moving Average** | **Unadjusted** | **PM_2.5_** | **Black Carbon** | **Particle Number** | **Log β Activity** |
| --- | --- | --- | --- | --- | --- | --- |
| **Sunspots(#)** | 0 | 1.12(0.85,1.39) | 1.06(0.79,1.33) | 1.10(0.83,1.38) | 0.78(0.45,1.11) | 0.96(0.69,1.24) |
|  | 1 | 1.15(0.88,1.42) | 1.09(0.81,1.36) | 1.14(0.86,1.41) | 0.81(0.48,1.15) | 1.00(0.72,1.27) |
|  | 7 | 1.25(0.97,1.53) | 1.18(0.90,1.46) | 1.23(0.94,1.52) | 0.93(0.58,1.28) | 1.08(0.79,1.36) |
|  | 14 | 1.35(1.06,1.64) | 1.28(0.99,1.57) | 1.33(1.03,1.62) | 1.05(0.69,1.42) | 1.16(0.87,1.46) |
|  | 21 | 1.43(1.13,1.73) | 1.35(1.05,1.66) | 1.41(1.10,1.72) | 1.15(0.77,1.53) | 1.23(0.93,1.54) |
|  | 28 | 1.45(1.14,1.75) | 1.37(1.06,1.67) | 1.43(1.12,1.74) | 1.17(0.78,1.56) | 1.24(0.93,1.55) |
| **IMF(nT)** | 0 | 2.96(1.80,4.11) | 2.75(1.59,3.91) | 2.97(1.80,4.15) | 2.41(1.24,3.59) | 2.26(1.09,3.42) |
|  | 1 | 4.53(3.17,5.89) | 4.31(2.95,5.68) | 4.56(3.17,5.94) | 3.68(2.27,5.08) | 3.70(2.32,5.07) |
|  | 7 | 10.20(8.10,12.30) | 9.80(7.68,11.92) | 10.17(8.03,12.32) | 8.64(6.35,10.92) | 8.69(6.53,10.86) |
|  | 14 | 13.65(11.27,16.04) | 13.19(10.78,15.60) | 13.63(11.18,16.07) | 11.91(9.21,14.61) | 11.90(9.42,14.38) |
|  | 21 | 14.78(12.24,17.32) | 14.33(11.76,16.90) | 14.67(12.07,17.27) | 13.07(10.15,15.98) | 12.92(10.28,15.57) |
|  | 28 | 15.25(12.66,17.84) | 14.72(12.10,17.34) | 15.19(12.54,17.84) | 13.62(10.62,16.62) | 13.32(10.61,16.03) |
| **Kp Index*** | 0 | 0.18(0.13,0.23) | 0.18(0.13,0.23) | 0.18(0.13,0.23) | 0.17(0.12,0.22) | 0.15(0.10,0.20) |
|  | 1 | 0.22(0.17,0.28) | 0.22(0.16,0.27) | 0.22(0.16,0.28) | 0.21(0.15,0.26) | 0.19(0.14,0.25) |
|  | 7 | 0.39(0.31,0.47) | 0.38(0.30,0.46) | 0.38(0.30,0.47) | 0.34(0.26,0.43) | 0.34(0.26,0.43) |
|  | 14 | 0.51(0.41,0.60) | 0.50(0.40,0.59) | 0.51(0.41,0.60) | 0.44(0.34,0.54) | 0.46(0.36,0.55) |
|  | 21 | 0.54(0.43,0.64) | 0.52(0.42,0.63) | 0.53(0.43,0.63) | 0.47(0.36,0.58) | 0.48(0.37,0.58) |
|  | 28 | 0.57(0.46,0.68) | 0.56(0.45,0.66) | 0.56(0.45,0.67) | 0.50(0.39,0.61) | 0.51(0.40,0.62) |
