# Supplementary material for: The role of solar and geomagnetic activity in endothelial activation and inflammation in the NAS cohort
Source: PLoS One. 2022 Jul 26;17(7):e0268700. doi: 10.1371/journal.pone.0268700 (PMC9321765; doi:10.1371/journal.pone.0268700)
Supplement: S5 Table — (DOCX) [file pone.0268700.s005.docx]

Supplementary Information 5

**Table S5.** Percent change (estimate*IQR*100) of CRP associated per IQR increase (95% CI) of exposure variable

| **Exposure** | **Moving**  **Average** | **Unadjusted** | **PM_2.5_** | **Black Carbon** | **Particle Number** | **Log β Activity** |
| --- | --- | --- | --- | --- | --- | --- |
| **Sunspots(#)** | 0 | 1.04(0.29,1.79) | 1.04(0.28,1.79) | 1.19(0.43,1.95) | 1.23(0.30,2.15) | 0.95(0.20,1.71) |
|  | 1 | 1.08(0.32,1.83) | 1.08(0.31,1.84) | 1.22(0.45,1.98) | 1.26(0.33,2.19) | 0.99(0.23,1.75) |
|  | 7 | 1.30(0.52,2.09) | 1.29(0.50,2.08) | 1.43(0.64,2.23) | 1.63(0.64,2.61) | 1.22(0.43,2.02) |
|  | 14 | 1.31(0.50,2.12) | 1.28(0.46,2.11) | 1.43(0.61,2.25) | 1.75(0.70,2.79) | 1.24(0.42,2.06) |
|  | 21 | 1.34(0.50,2.18) | 1.32(0.48,2.17) | 1.46(0.61,2.31) | 1.79(0.70,2.88) | 1.26(0.41,2.11) |
|  | 28 | 1.34(0.49,2.19) | 1.34(0.48,2.20) | 1.46(0.60,2.32) | 1.76(0.66,2.86) | 1.27(0.40,2.13) |
| **IMF(nT)** | 0 | 0.36(-2.83,3.54) | 0.96(-2.43,4.36) | 1.35(-1.88,4.58) | 1.35(-2.18,4.88) | 0.20(-3.00,3.39) |
|  | 1 | 0.34(-3.43,4.10) | 0.79(-3.07,4.65) | 1.50(-2.31,5.31) | 1.02(-3.12,5.16) | 0.05(-3.74,3.83) |
|  | 7 | 4.73(-1.17,10.63) | 4.99(-1.01,11.00) | 6.43(0.45,12.41) | 6.09(-0.74,12.92) | 4.34(-1.65,10.34) |
|  | 14 | 6.98(0.22,13.74) | 7.45(0.55,14.35) | 8.69(1.83,15.54) | 8.84(0.73,16.94) | 6.25(-0.66,13.16) |
|  | 21 | 7.05(-0.15,14.24) | 7.70(0.32,15.07) | 8.84(1.53,16.15) | 7.70(-0.98,16.38) | 6.24(-1.12,13.61) |
|  | 28 | 8.03(0.68,15.38) | 9.24(1.67,16.82) | 9.81(2.36,17.27) | 8.70(-0.17,17.56) | 7.24(-0.30,14.77) |
| **Kp Index *** | 0 | -0.01(-0.15,0.13) | 0.02(-0.13,0.16) | 0.03(-0.11,0.17) | 0.04(-0.11,0.19) | -0.01(-0.15,0.13) |
|  | 1 | -0.01(-0.17,0.14) | 0.00(-0.16,0.16) | 0.02(-0.13,0.18) | 0.01(-0.16,0.18) | -0.02(-0.18,0.13) |
|  | 7 | 0.04(-0.19,0.27) | 0.07(-0.18,0.31) | 0.08(-0.15,0.32) | 0.04(-0.21,0.29) | 0.01(-0.23,0.25) |
|  | 14 | 0.10(-0.17,0.37) | 0.13(-0.15,0.41) | 0.15(-0.12,0.42) | 0.11(-0.19,0.40) | 0.06(-0.22,0.33) |
|  | 21 | 0.09(-0.20,0.38) | 0.15(-0.15,0.45) | 0.14(-0.15,0.44) | 0.07(-0.25,0.38) | 0.05(-0.24,0.34) |
|  | 28 | 0.17(-0.13,0.47) | 0.24(-0.07,0.55) | 0.23(-0.08,0.53) | 0.14(-0.19,0.47) | 0.13(-0.18,0.43) |
