# Supplementary material for: The role of solar and geomagnetic activity in endothelial activation and inflammation in the NAS cohort
Source: PLoS One. 2022 Jul 26;17(7):e0268700. doi: 10.1371/journal.pone.0268700 (PMC9321765; doi:10.1371/journal.pone.0268700)
Supplement: S6 Table — (DOCX) [file pone.0268700.s006.docx]

Supplementary Information 6

**Table S6.** Percent change (estimate*IQR*100) of Fibrinogen associated per IQR increase (95% CI) of exposure variable

| **Exposure** | **Moving Average** | **Unadjusted** | **PM_2.5_** | **Black Carbon** | **Particle Number** | **Log β Activity** |
| --- | --- | --- | --- | --- | --- | --- |
| **Sunspots(#)** | 0 | 0.09(-0.11,0.29) | 0.13(-0.08,0.34) | 0.08(-0.13,0.28) | 0.36(0.12,0.60) | 0.13(-0.08,0.33) |
|  | 1 | 0.09(-0.11,0.30) | 0.13(-0.08,0.34) | 0.08(-0.13,0.29) | 0.35(0.11,0.59) | 0.13(-0.07,0.34) |
|  | 7 | 0.13(-0.08,0.34) | 0.19(-0.02,0.41) | 0.12(-0.09,0.34) | 0.41(0.16,0.66) | 0.18(-0.03,0.39) |
|  | 14 | 0.12(-0.10,0.33) | 0.18(-0.04,0.40) | 0.11(-0.12,0.33) | 0.42(0.16,0.68) | 0.16(-0.06,0.38) |
|  | 21 | 0.09(-0.13,0.31) | 0.15(-0.08,0.38) | 0.08(-0.15,0.31) | 0.40(0.13,0.68) | 0.13(-0.09,0.36) |
|  | 28 | 0.09(-0.13,0.32) | 0.15(-0.08,0.38) | 0.07(-0.16,0.31) | 0.40(0.13,0.68) | 0.14(-0.09,0.37) |
| **IMF(nT)** | 0 | 0.43(-0.40,1.27) | 0.27(-0.70,1.24) | 0.35(-0.50,1.20) | 0.65(-0.25,1.55) | 0.48(-0.36,1.32) |
|  | 1 | 0.03(-0.96,1.03) | -0.08(-1.14,0.99) | -0.07(-1.08,0.94) | 0.29(-0.77,1.34) | 0.11(-0.89,1.11) |
|  | 7 | -0.34(-1.89,1.21) | -0.32(-1.97,1.33) | -0.51(-2.08,1.06) | 1.09(-0.64,2.82) | -0.12(-1.71,1.46) |
|  | 14 | -1.08(-2.84,0.68) | -0.98(-2.86,0.89) | -1.30(-3.10,0.49) | 0.73(-1.30,2.76) | -0.84(-2.66,0.98) |
|  | 21 | -1.14(-3.01,0.73) | -0.99(-2.99,1.01) | -1.37(-3.27,0.54) | 0.76(-1.39,2.92) | -0.90(-2.83,1.03) |
|  | 28 | -0.88(-2.78,1.03) | -0.69(-2.73,1.35) | -1.12(-3.07,0.82) | 0.98(-1.23,3.18) | -0.61(-2.58,1.36) |
| **Kp Index*** | 0 | -0.02(-0.06,0.01) | -0.03(-0.07,0.01) | -0.02(-0.06,0.01) | -0.02(-0.05,0.02) | -0.02(-0.05,0.02) |
|  | 1 | -0.04(-0.08,0.00) | -0.04(-0.08,0.00) | -0.04(-0.08,0.00) | -0.03(-0.07,0.01) | -0.03(-0.07,0.01) |
|  | 7 | -0.08(-0.14,-0.02) | -0.09(-0.16,-0.03) | -0.09(-0.15,-0.03) | -0.05(-0.11,0.01) | -0.08(-0.14,-0.01) |
|  | 14 | -0.09(-0.16,-0.02) | -0.10(-0.17,-0.02) | -0.09(-0.16,-0.02) | -0.05(-0.12,0.02) | -0.08(-0.15,-0.01) |
|  | 21 | -0.09(-0.17,-0.02) | -0.10(-0.18,-0.02) | -0.10(-0.17,-0.02) | -0.06(-0.14,0.01) | -0.09(-0.16,-0.01) |
|  | 28 | -0.10(-0.18,-0.02) | -0.10(-0.19,-0.02) | -0.10(-0.18,-0.03) | -0.07(-0.15,0.01) | -0.09(-0.17,-0.02) |
